# Predominantly lithogenic chromium and nickel elevate sediment guideline quotients and exposure screening in a subtropical African water supply reservoir

**DOI:** 10.1007/s10653-026-03558-1

**Published:** 2026-10-26

**Authors:** Tatenda Dalu, Farai Dondofema, Chad Keates, Linton F. Munyai

**Affiliations:** 1 Centre for Invasion Biology, School for Climate Studies, Stellenbosch University, Stellenbosch, South Africa; 2 Department of Geography and Environmental Sciences, University of Venda, Thohoyandou, South Africa; 3 South African Institute for Aquatic Biodiversity, Makhanda, 6140 South Africa; 4 The Wilderness Project Foundation, Johannesburg, South Africa; 5 Aquatic Systems Research Group, Department of Water and Sanitation, University of Limpopo, Polokwane, South Africa

**Keywords:** Potentially toxic elements, Reservoir sediment, Geogenic control, Sediment quality guidelines, Human health screening, Luvuvhu river

## Abstract

Reservoir sediments accumulate potentially toxic elements from both catchment weathering and human activity, and concentration-based indices regularly confound the two. We evaluated this distinction in the Nandoni Reservoir, a water supply impoundment along the Luvuvhu River, South Africa. Surface sediment (0 to 5 cm) was collected using an Ekman grab at 21 sampling sitessite in October 2023, at the end of the dry season, and 26 elements were quantified by inductively coupled plasma mass spectrometry (ICP–MS). Chromium, nickel, copper and lead covaried strongly with aluminium, with element to aluminium ratio coefficients of variation of 0.36 or less. Aluminium normalised enrichment factors for Pb, Zn, Cd, As and Hg were at or below crustal proportions, whereas Cr, Cu and Ni were elevated relative to average shale. These patterns are consistent with predominantly lithogenic control, but do not by themselves establish source dominance and do not exclude diffuse anthropogenic inputs. Chromium exceeded the consensus probable effect concentration at 14 sampling sitesite and nickel at 13, and the mean probable effect concentration quotient ranged from 0.18 to 0.85. Exceedance of this kind indicates screening level concern rather than demonstrated ecological effect. Deterministic hazard indices were 0.07 to 0.34 for an adult maintenance worker and 0.36 to 1.67 for a child with incidental shoreline contact, exceeding one at nine sites for the child, falling to three sites when chromium was treated as Cr(III) rather than as the conservative upper bound Cr(VI). Replacing average shale with a pre–industrial reference inflated the pollution load index 4.0 fold, driven almost entirely by Ni, Cu and Cr. Source attribution, ecological screening and exposure assessment must be reported as separate lines of evidence.

**Supplementary Information:**

The online version contains supplementary material available at https://doi.org/10.1007/s10653-026-03558-1.

## Introduction

Trace element contamination of aquatic sediments is a global environmental concern arising from both lithogenic weathering and anthropogenic inputs (Nawrot et al., 2021; Sigue et al., 2025; Yiika et al., 2024). Reservoir construction interrupts longitudinal sediment transport and creates depositional zones where fine particles and potentially toxic elements accumulate (Dong et al., 2019; Sojka et al., 2018). These deposits preserve information about catchment inputs, but they can also become secondary sources during drawdown, resuspension, erosion of exposed margins and sediment handling (Eggleton & Thomas, 2004; Bing et al., 2022; Fremion et al., 2016). The relevant concern is therefore not only whether an element is elevated relative to a reference background, but also whether its concentration, chemical form and exposure pathway could affect benthic organisms, wildlife and humans.

The conventional assessment strategy combines three analytically distinct components, and each answers a different question (Long et al., 1995; US EPA, 1989, 2004; MacDonald et al., 2000; Wojciechowska et al., 2019; Varol, 2020; Zhu et al., 2019; Canpolat et al., 2022; Sojka et al., 2022a, 2022b). First, four contamination indices measure departure from an assumed background. The contamination factor expresses the measured concentration of a single element as a ratio to a selected geochemical background (Hakanson, 1980). The geo–accumulation index expresses the same comparison on a base–two logarithmic scale, with a multiplier of 1.5 applied to the background to accommodate natural lithological variability (Muller, 1969). The pollution load index is the geometric mean of the contamination factors of the elements considered (Tomlinson et al., 1980). The potential ecological risk index sums the contamination factors after weighting each element by a toxic response factor (Hakanson, 1980). Second, sediment quality guidelines are empirical concentration benchmarks associated with a probability of biological effects, derived by matching paired datasets of sediment chemistry and biological response (Burton, 2002; Long et al., 1995; MacDonald et al., 2000). Third, exposure models estimate risk under specified assumptions about contact, uptake and toxicity. Treating these outputs as interchangeable produces two opposite errors. A sediment whose composition is set by the parent rock may return a high contamination factor and be targeted for source control that cannot reduce a concentration the geology supplies, or an element recognised as geogenic may be dismissed as harmless even though it exceeds an effects–based benchmark and reaches a nearby receptor (Burton, 2002; Dong et al., 2019; Eggleton & Thomas, 2004).

This problem arises wherever local geology, weathering intensity, particle sorting and hydrological connectivity exert strong control on sediment composition, which is a global rather than a regional condition (Dalu et al., 2018; Tiabou et al., 2025). Its practical consequences are greatest where monitoring records are short and locally derived geochemical baselines are scarce, because a generic external background is then adopted by default (Birch, 2017; Coral et al., 2022; Nawrot et al., 2021; Reimann & de Caritat, 2005; Rudnick & Gao, 2003). Aluminium normalisation and regional geochemical surveys help identify whether an element varies independently of the detrital matrix, but aluminium is not universal proof of natural origin (Burton, 2002; Reimann & de Caritat, 2005; Schropp et al., 1990). It varies with mineralogy and grain size, and a diffuse anthropogenic input may remain correlated with the same fine fraction. Source attribution should therefore be expressed as the strength of evidence for lithogenic control rather than as a definitive finding.

Nandoni Reservoir impounds the Luvuvhu River in Limpopo Province, South Africa. Its catchment includes sandstone and basaltic units of the Soutpansberg Group, granitic gneiss of the Limpopo Belt, communal rangeland, agriculture, plantation forestry and dense settlement (Barker et al., 2006; Edokpayi et al., 2022; Shibambu et al., 2025). Unsewered settlements, unpaved roads and agricultural runoff provide plausible diffuse sources, while the regional geology provides a plausible natural source of chromium, nickel and other lithophile elements. Previous work in the catchment has reported trace elements in tributary sediments and water quality concerns (Barnhoorn et al., 2009; Dahms et al., 2017; Edokpayi et al., 2022; Madilonga et al., 2021; Shibambu et al., 2025). Those studies were confined to riverine reaches, reported contamination indices against a single external background, and did not evaluate whether their index classifications would survive a change of reference composition. None assessed the impounded basin itself, where hydraulic sorting concentrates the fine fraction that carries most trace elements. The specific contribution of the present study is therefore to combine, on one dataset, four elements of evidence that are normally reported in isolation: aluminium normalised source diagnostics, consensus effects based sediment quality benchmarks, deterministic exposure screening for two contrasting receptors, and a quantified sensitivity analysis of the composite indices to the choice of external background, including identification of which individual elements drive that sensitivity.

We investigated whether the spatial distribution of potentially toxic elements in Nandoni Reservoir is more consistent with variation in the detrital matrix than with independent anthropogenic loading, and whether source attribution changes the ecological and human health screening conclusion. We quantified 26 elements across the depositional gradient, evaluated aluminium normalised associations and enrichment, compared priority elements with consensus sediment quality benchmarks, estimated deterministic exposure screening values for two contrasting receptors, tested the association between water depth and element concentrations, and quantified the sensitivity of composite indices to two external reference sets (Muller, 1969; Hakanson, 1980; Tomlinson et al., 1980; Birch, 2017; Reimann & de Caritat, 2005). The two receptors were an adult maintenance worker, assumed to handle sediment during dredging and intake maintenance, and a child, assumed to have incidental contact with exposed sediment on the reservoir margin during low water periods. Incidental shoreline contact therefore applies to the child scenario only, whereas the worker scenario represents occupational handling of recovered sediment and not shoreline recreation. We evaluated three linked predictions: (i) predominantly lithogenic control would be indicated by strong monotonic associations with Al, relatively stable element to Al ratios and little or no enrichment of Pb, Zn, Cd, As and Hg, whereas an independent source would be indicated by decoupling from Al, noting that association with Al cannot by itself separate lithogenic material from anthropogenic input transported with the same fine fraction, (ii) effects based and exposure based screening could identify concern even where enrichment indices suggested limited anthropogenic loading, because total concentration, effects based benchmarks and modelled exposure are not equivalent measures, and (iii) composite indices would be sensitive to the selected background, with no basis for treating a single generic background as an intrinsic property of the reservoir.

## Materials and methods

### Study area

**Fig. 1:**
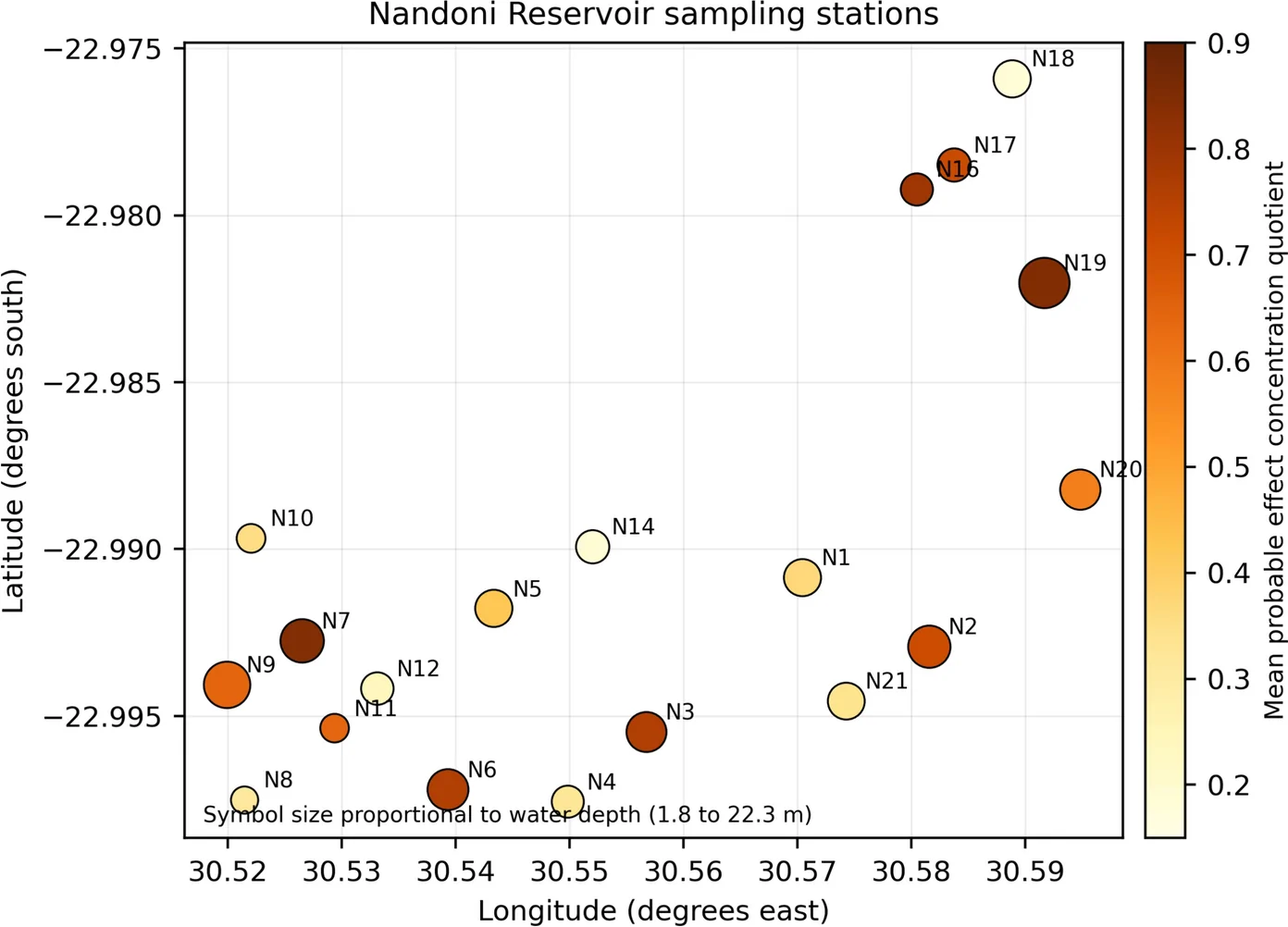
Nandoni Reservoir sampling sites. Symbol colour indicates the mean probable effect concentration quotient, and symbol size is proportional to water depth at the site. The figure describes spatial screening patterns during the October 2023 survey and does not imply temporal persistence

Nandoni Reservoir impounds the Luvuvhu River in Limpopo Province, South Africa (22.99° S, 30.55° E) (Fig. 1). The river rises in the Soutpansberg Mountains and flows for approximately 200 km before joining the Limpopo River at Crooks Corner in Kruger National Park (Barker et al., 2006; Edokpayi et al., 2022). Mean annual precipitation across the catchment is approximately 608 mm, with most rainfall between October and March. Rainfall increases from about 450 mm in the low lying eastern sector to approximately 2300 mm on the Soutpansberg escarpment. The catchment is underlain principally by sandstone and basaltic units of the Soutpansberg Group, together with granitic gneiss of the Limpopo Belt. Land use comprises mainly communal rangeland, subsistence and irrigated agriculture, plantation forestry and dense unsewered settlement.

Twenty one sites were sampled on a single occasion in October 2023, at the end of the dry season and before the main summer inflow period. Sites were allocated by stratified purposive design rather than at random: the impoundment was divided into riverine, transitional and lacustrine zones, and sites were distributed within each zone to span the full range of accessible water depth (1.8 to 22.3 m) and both shorelines, giving spatial coverage of the whole basin rather than of the dam wall alone (Fig. 1). Purposive allocation was chosen because the study objective was to characterise the depositional gradient, which is structured rather than spatially random. The design does not support design based inference to unsampled locations. Surface sediment was collected with an Ekman grab (Royal Eijkelkamp; capacity of 3.5 L, dimensions 152 × 152 × 152 mm). At each sitesite 3 grab deployments were made, the upper 0 to 5 cm of undisturbed surface sediment was retained from each, and the retained material was combined into a single composite sample per site and homogenised with an acid washed polypropylene scoop before subsampling. Grabs in which the sediment surface appeared disturbed or the jaws failed to close fully were discarded and the deployment repeated. The grab and all sampling implements were washed with ambient reservoir water and rinsed with deionised water between sites. Composites were transferred to acid washed containers and stored at 4 °C in the dark until analysis. Water depth was measured with an echo sounder (Garmin ECHOMAP UHD2 Series), and site position was recorded with a handheld global positioning system receiver (Garmin GPSMAP 64S). An Ekman grab was selected in preference to a Van Veen grab because the target substrate is the soft, fine grained, recently deposited material of a standing water reservoir, and the Ekman design is lightweight, deployable from a small boat without a winch, and closes with minimal bow wave, which preserves the flocculent surface layer that carries most of the trace element load. Van Veen grabs are heavier, are intended for firmer or coarser marine and estuarine substrates, and would have required vessel mounted lifting gear not available at Nandoni. Constraints on sampling should be noted, as sampling was restricted to one hydrological period, so seasonal variability was not characterised. A single composite per site does not quantify within site heterogeneity, and very shallow marginal areas and areas obstructed by standing dead trees could not be safely accessed by boat and are therefore under represented.

### Analysis and quality control

Samples were oven dried at 60 °C to constant mass, disaggregated with an agate mortar and pestle and passed through a 2 mm nylon sieve. The analytical suite comprised 26 elements: Na, Mg, P, K, Ca, B, Al, Si, V, Cr, Mn, Fe, Co, Ni, Cu, Zn, As, Se, Sr, Mo, Cd, Sn, Sb, Ba, Hg and Pb. Sample preparation and determination were carried out by the Central Analytical Facility of Stellenbosch University, a laboratory accredited by the South African National Accreditation System. A representative 0.50 g aliquot of the sieved fraction was placed in a closed microwave digestion vessel with 9 mL of concentrated nitric acid followed by 3 mL of concentrated hydrochloric acid. The vessel was heated to 175 ± 5 °C over 5.5 ± 0.25 min and held at that temperature for 4.5 min. After cooling, the digest was clarified and diluted to a recorded final volume with reagent water. Elemental concentrations were determined by inductively coupled plasma mass spectrometry (ICP–MS) following US EPA Method 6020B. This digestion recovers the total or pseudo–total element burden rather than the freely dissolved or operationally bioavailable fraction, and all contamination indices, guideline comparisons and exposure estimates reported here are therefore based on pseudo–total concentrations. Concentrations are reported on a dry mass basis as mg kg^−1^.

Quality control comprised reagent blanks, certified reference material and analytical duplicates. Two reagent blanks were digested and analysed with each batch, and blank concentrations were below the limit of quantification for all reported trace elements. Limits of detection were determined as three times, and limits of quantification as ten times, the standard deviation of the reagent blanks, and element specific values are reported in Table S1. Accuracy was assessed against certified reference material WQB–1 analysed in five replicates. Recoveries for the priority elements were Cr 108.7%, Ni 81.3%, Cu 103.7%, Zn 102.5%, As 109.5%, Cd 96.2%, Pb 100.8% and Hg 111.4%, with relative standard deviations of 2.4% to 4.8% (Table S1). Recoveries for the remaining reported elements fell within 83.7% to 107.8%, with the single exception of Sb, whose replicate relative standard deviation of 22.2% indicates poor analytical precision at the concentrations encountered. Antimony is therefore reported descriptively only and is excluded from interpretation. Three sites, N7, N16 and N19, were sampled and analysed in duplicate, and duplicate agreement was within 6% for all reported elements. Values below the limit of quantification were treated as missing rather than substituted. Censoring was negligible for the elements that carry the interpretation: Cr, Ni, Cu, Zn, As, Cd, Pb, Co, V, Mn and Ba were quantified at all 21 sites (0% censored). Censoring was material for three elements only, namely Hg (14 of 21 sites censored, 67%), Na (12 of 21, 57%) and Se (4 of 21, 19%). Because the censoring rate for Hg and Na exceeds 50%, neither a substitution rule nor a maximum–likelihood censored estimator would yield a defensible distribution, and both elements were therefore excluded from index calculation and retained for descriptive reporting only. Selenium, at 19% censoring, is similarly reported descriptively and is not used in any index. For all elements used in the indices and risk calculations, no observations were censored, so the choice of censored–data method does not affect any reported result.

### Source partitioning

Aluminium was used as a conservative reference element because it is commonly associated with the aluminosilicate detrital fraction (Burton, 2002; Reimann & de Caritat, 2005; Schropp et al., 1990). For each element, two complementary diagnostics were calculated. Spearman’s rank correlation coefficient (ρ) is a non–parametric measure of monotonic association that ranges from − 1 to + 1 and is computed on the ranks rather than the raw values, so it is insensitive to the positively skewed distributions and occasional extreme values typical of sediment geochemical data. It was used here to quantify monotonic covariation between each element and Al. The coefficient of variation of the element to Al ratio, calculated as the standard deviation of the ratio divided by its mean across sites, quantified the stability of that association. A low coefficient of variation indicates that the element maintains an approximately constant proportion to the detrital matrix across the basin. These diagnostics describe matrix control and are not, on their own, a source apportionment model. An anthropogenic input transported as fine particles could remain correlated with Al and would not be distinguished by either diagnostic. Evidence for lithogenic control was therefore evaluated jointly with enrichment factors, geo–accumulation values, the spatial pattern and the known catchment geology, and is reported throughout as the strength of evidence rather than as demonstrated source attribution.

The aluminium normalised enrichment factor was calculated as EF_i_ = (C_i_/Al)_sample_/(C_i_/Al)_reference_, using average shale (Turekian & Wedepohl, 1961) as the external reference composition. Values of 0.5 to 2 were interpreted as broadly consistent with reference proportions, 2 to 5 as moderate enrichment and values above 5 as substantial enrichment, following the screening convention of Sutherland (2000). These categories are descriptive and should not be treated as universal source thresholds. The enrichment factor analysis was used to identify elements that depart from the detrital matrix, not to establish the absolute absence of anthropogenic inputs.

### Contamination and ecological risk indices

The contamination factor was calculated as CFi = Ci/Bi, where Ci is the measured concentration and Bi is the selected background. The pollution load index was calculated as PLI = (CF1 × CF2 × CF3 … × CFn)1/n, where n is the number of elements considered, and the potential ecological risk index as PERI = Σ(Ti × CFi), where Ti is the toxic response factor. The toxic response factors applied were 1 for Zn, 2 for Cr, 5 for Cu and Pb, 6 for Ni and 30 for Cd. Of these, the value of 6 for Ni follows Sojka et al. (2022b) rather than the original value of 5 given by Hakanson (1980) and is adopted here for comparability with that framework. Both indices were calculated over the same six elements (Cd, Cu, Cr, Ni, Pb and Zn) under both background sets, so that the two reference compositions are compared on an identical element basis. Calculations were repeated using average shale (Turekian & Wedepohl, 1961) and the lower pre–industrial reference values reported by Sojka et al. (2022b). Both are external comparators rather than locally measured natural baselines, and the sensitivity analysis therefore tests how far the resulting classification depends on reference choice, not which background is correct for Nandoni.

### Sediment quality guidelines

Measured concentrations were compared with the consensus threshold effect concentrations (TEC) and probable effect concentrations (PEC) reported by MacDonald et al. (2000), which build on the effects range approach of Long et al. (1995). The mean PEC quotient was calculated as the arithmetic mean of Ci/PECi across As, Cd, Cr, Cu, Ni, Pb, Zn and Hg. These eight elements were used because they are the only elements in the 26 element analytical suite for which MacDonald et al. (2000) published consensus based freshwater TEC and PEC values. The remaining 18 elements, including Co, V, Mn and Ba, have no consensus freshwater benchmark and could not be included without introducing a benchmark from a different derivation framework, which would make the quotient internally inconsistent. Those elements are nevertheless retained in the human health screening, where toxicity reference values do exist. These benchmarks are screening tools derived from empirical effects databases, and exceedance indicates an increased likelihood of adverse effects within the derivation dataset rather than a demonstrated effect at Nandoni reservoir. The absence of grain size, total organic carbon, acid volatile sulphide and pore water measurements does not invalidate the use of consensus PECs, which were derived from total sediment concentrations and are intended for precisely this screening purpose. It does, however, limit site specific interpretation, because partitioning and bioavailability at Nandoni reservoir cannot be evaluated.

### Human health risk assessment

Average daily doses from incidental ingestion of sediment and from dermal contact were calculated using the United States Environmental Protection Agency framework (US EPA, 1989, 2004, 2011, 2026). For ingestion, ADDing = C × IR × EF × ED/(BW × AT) × 10^−6^. For dermal contact, ADDderm = C × SA × AF × ABS × EF × ED/(BW × AT) × 10^−6^, where C is concentration in mg kg^−1^, IR is ingestion rate in mg day^−1^, EF is exposure frequency in days year^−1^, ED is exposure duration in years, BW is body mass in kg, AT is averaging time in days, SA is exposed skin area in cm^2^, AF is the sediment adherence factor in mg cm^−2^ and ABSd is the dermal absorption fraction. The adult maintenance worker scenario used IR = 100 mg day^−1^, EF = 180 days year^−1^, ED = 35 years, BW = 70 kg, SA = 3 300 cm^2^ and AF = 0.2 mg cm^−2^. The child incidental contact scenario used IR = 200 mg day^−1^, EF = 104 days year^−1^, ED = 6 years, BW = 15 kg, SA = 2 800 cm^2^ and AF = 0.2 mg cm^−2^. The non-carcinogenic averaging time was ED × 365 days and the carcinogenic averaging time was 70 × 365 days. Oral reference doses and oral slope factors are not directly applicable to the dermal route, and were not applied to it unadjusted. Following US EPA (2004), route specific dermal toxicity values were derived by multiplying the oral reference dose by the gastrointestinal absorption fraction for each element, so that the dermal hazard quotient was calculated as ADDderm/(RfDo × ABSgi) while the ingestion hazard quotient was calculated as ADDing/RfDo. Hazard quotients for the two pathways were computed separately, are reported separately as pathway contributions, and were summed across pathways and elements only to produce the hazard index. Incremental lifetime cancer risk was calculated for Cr, Ni, As, Cd and Pb as the lifetime average daily dose from ingestion multiplied by the oral slope factor, and was summed across those elements. Because validated dermal slope factors are not available for these elements, cancer risk is reported for the ingestion pathway only, which is the dominant pathway for all five, and the dermal contribution to cancer risk is therefore not estimated rather than being assumed to be zero. Toxicity values were taken from the cited US EPA screening resources. Because the analytical method determines total Cr and not its oxidation state, the primary calculation conservatively treats all measured Cr as Cr(VI), which maximises both the chromium hazard quotient and the chromium cancer risk. Since this single assumption governs a large share of the estimated risk, a parallel sensitivity calculation was run in which all measured Cr was instead treated as Cr(III), using the oral reference dose for Cr(III) of 1.5 mg kg^−1^ day^−1^ and applying no slope factor, Cr(III) not being classified as a carcinogen by the oral route. The two calculations bracket the true value and are reported together. All results are deterministic screening estimates and are not probability distributions or observed disease risks.

### Data analysis

**Fig. 2:**
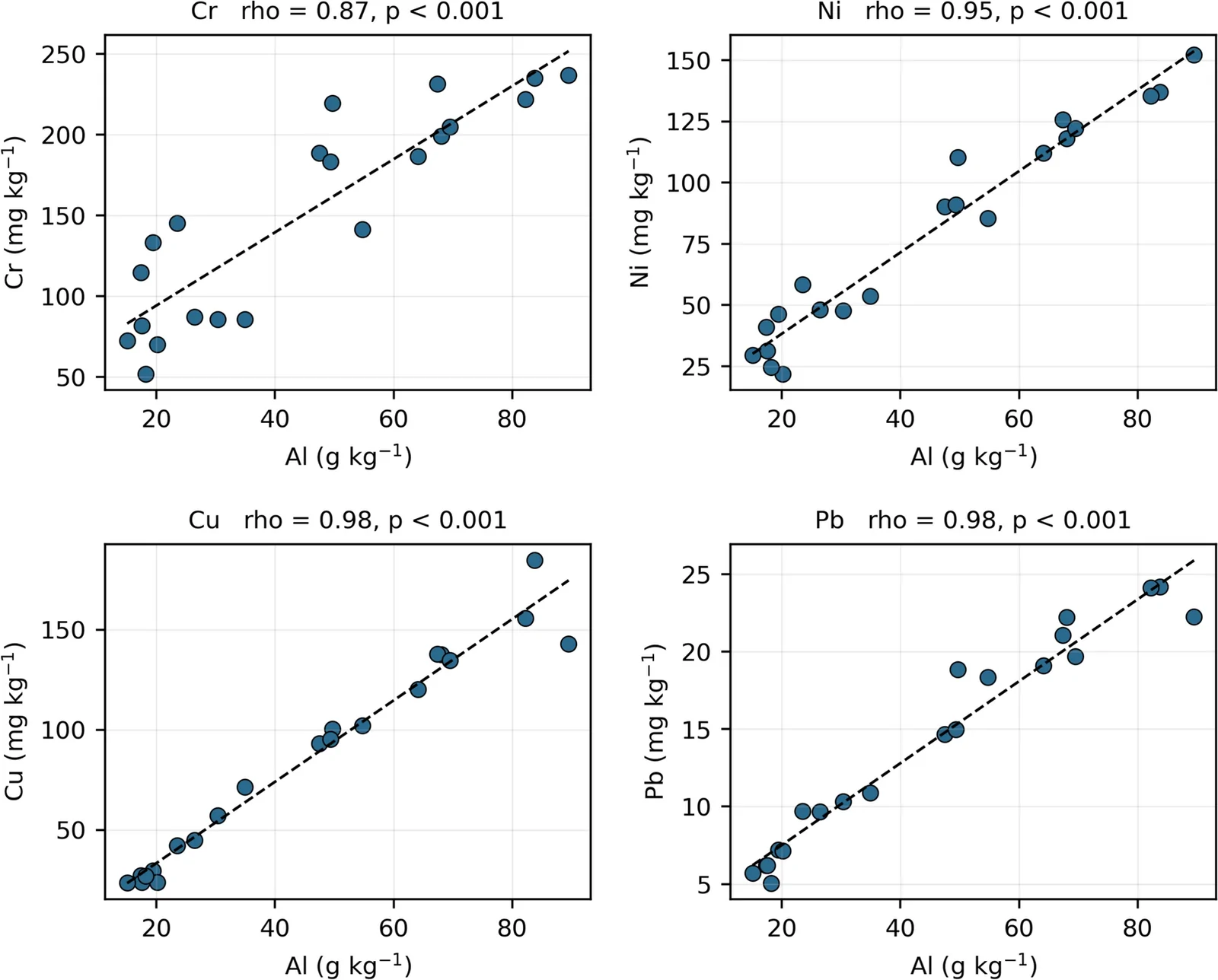
Concentrations of Cr, Ni, Cu and Pb against Al across the 21 sites. Dashed ordinary least squares lines are shown for visualisation, while inference was based on Spearman rank correlations. The associations are consistent with a shared detrital control

All analyses were conducted in Python 3.12 using pandas, SciPy and Matplotlib. Spearman rank correlations were two sided and calculated using pairwise quantified observations. The reported p values are descriptive because the study tests multiple element and depth associations and the sample size is 21 sites, and no correction for multiple comparison was applied. Water depth associations were evaluated with Spearman correlation and are interpreted as associations rather than causal effects. Ordinary least squares lines in Fig. 2 are visual summaries only. Multivariable models were precluded because grain size, organic carbon, redox conditions and other potential confounders were not measured. Indices and exposure estimates were calculated site by site using the same measured concentrations and are reported as ranges, means or counts of sites. To separate background uncertainty from contamination evidence, the contribution of each element to the difference between the two background dependent index sets was decomposed analytically. For the pollution load index, which is a geometric mean of ratios, the inflation factor on substituting background set B2 for B1 is the geometric mean of the element wise background ratios B1i/B2i and is therefore identical at every site and independent of the measured concentrations. For the potential ecological risk index, the contribution of each element to the change was calculated as Ti × Ci ×(1/B2i – 1/B1i) and expressed as a percentage of the total change.

## Results

### Elemental composition

Iron and aluminium dominated the measured elemental inventory, with ranges of 21 456 to 110 145 mg kg^−1^ for Fe and 15 064 to 89 525 mg kg^−1^ for Al (Table 1). Among the priority elements, mean concentrations increased in the order Cd < As < Pb < Zn < Ni < Cu < Cr. Chromium ranged from 51.9 to 236.8 mg kg^−1^ and nickel from 21.8 to 152.2 mg kg^−1^. Cadmium ranged from 0.010 to 0.064 mg kg^−1^, arsenic did not exceed 3.73 mg kg^−1^ and lead ranged from 5.1 to 24.2 mg kg^−1^. Mercury was quantified at 7 sites and did not exceed 0.073 mg kg^−1^ at quantified sites. The spatial pattern was broadly shared among the matrix associated elements. Sites N7, N16, N17 and N19 generally had the highest concentrations, while N13, N14 and N18 generally had the lowest. Concentrations therefore varied by approximately fivefold between the lowest and highest sites, but the rank order was similar for Cr, Ni, Cu, Zn, Pb, Co, V, Mn, As, Cd, Ba and Sn. This common spatial structure is consistent with changes in the amount or composition of deposited detrital material, although the available measurements do not distinguish particle size sorting from mineralogical variation.

**Table 1:** Descriptive statistics for elements in Nandoni Reservoir surface sediment sampled in October 2023. Concentrations are dry mass concentrations in mg kg^−1^. n is the number of quantified observations. Element specific limits of detection and quantification, certified reference material recoveries and censoring rates are given in Table S1

Element	*n*	Mean	SD	Minimum	Median	Maximum
Al	21	45,175	25,039	15,064	47,479	89,525
Fe	21	58,631	29,231	21,456	63,760	110,145
Mn	21	1063	693	317	858	2858
Ba	21	216	135	55.2	181	517
Cr	21	151	63.8	51.9	145	237
V	21	119	56.1	42	130	215
Cu	21	84.5	51.8	23.6	93.2	185
Ni	21	80.1	42.6	21.8	85.4	152
Zn	21	59.5	31.1	15.8	63.6	109
Sr	21	33.7	18.3	14.7	30.3	98.2
Co	21	26.2	11.1	9.54	28.3	42.6
Pb	21	14.2	6.78	5.05	14.7	24.2
As	21	1.78	1.01	0.507	1.89	3.73
Sn	21	1.25	0.623	0.493	1.14	2.33
Se	17	0.834	0.336	0.168	0.892	1.36
Mo	21	0.162	0.066	0.061	0.149	0.354
Sb	21	0.074	0.029	0.014	0.073	0.121
Hg	7	0.04	0.015	0.03	0.034	0.073
Cd	21	0.036	0.019	0.01	0.037	0.064

### Source partitioning and evidence for lithogenic control

Element concentrations generally covaried positively with Al (Table 2, Fig. 2). Spearman ρ was 0.98 for Pb and Cu, 0.95 for Ni and Zn, 0.94 for As and Co, 0.90 for Cd and 0.87 for Cr, with *p *< 0.001 in every case. Antimony and Hg showed weak associations with Al, while Sr showed a moderate association (ρ = 0.46). These exceptions should not be read as evidence of an independent source, because Sb had a replicate relative standard deviation of 22.2%, Hg was quantified at only 7 sites, and Sr is controlled by carbonate and feldspar mineralogy that differs from the processes controlling aluminosilicates. For the elements most relevant to the interpretation, coefficients of variation of element to Al ratios were 0.13 for Pb and V, 0.15 for Cu and Zn, 0.18 for Ni and As, 0.21 for Cd and 0.36 for Cr. The strong Al associations and relatively stable element to Al ratios are consistent with predominantly matrix controlled distributions across the reservoir. They cannot, however, by themselves distinguish lithogenic inputs from diffuse anthropogenic contributions associated with the fine sediment fraction, and they are reported here as evidence against a strong, spatially discrete anthropogenic input rather than as evidence that no anthropogenic input exists.

**Table 2:** Source oriented diagnostics for the principal elements. Spearman ρ is the rank correlation with Al, CV is the coefficient of variation of the element to Al ratio, EF is the aluminium normalised enrichment factor relative to average shale, and Igeo is the geo–accumulation index. Diagnostics describe matrix association and do not by themselves establish source

Element	ρ with Al	*p*	CV of element to Al	Enrichment factor range	Sites with Igeo above 0
Pb	0.98	< 0.001	0.13	0.99 to 1.65	0
Cu	0.98	< 0.001	0.15	2.11 to 3.92	12
V	0.96	< 0.001	0.13	1.40 to 2.17	2
Zn	0.95	< 0.001	0.15	0.66 to 1.42	0
Ni	0.95	< 0.001	0.19	1.28 to 2.92	8
Ba	0.95	< 0.001	0.17	not calculated	0
Sn	0.95	< 0.001	0.17	not calculated	0
As	0.94	< 0.001	0.18	0.17 to 0.34	0
Co	0.94	< 0.001	0.24	1.89 to 4.39	10
Cd	0.9	< 0.001	0.21	0.16 to 0.34	0
Cr	0.87	< 0.001	0.36	2.18 to 6.11	12
Mn	0.85	< 0.001	0.37	1.32 to 4.62	7
Se	0.81	< 0.001	0.25	not calculated	0
Mo	0.51	0.017	0.59	not calculated	0
Sr	0.46	0.034	0.96	not calculated	0
Hg	0.21	0.645	0.28	0.07 to 0.16	0
Sb	0.1	0.654	0.71	not calculated	0

**Fig. 3:**
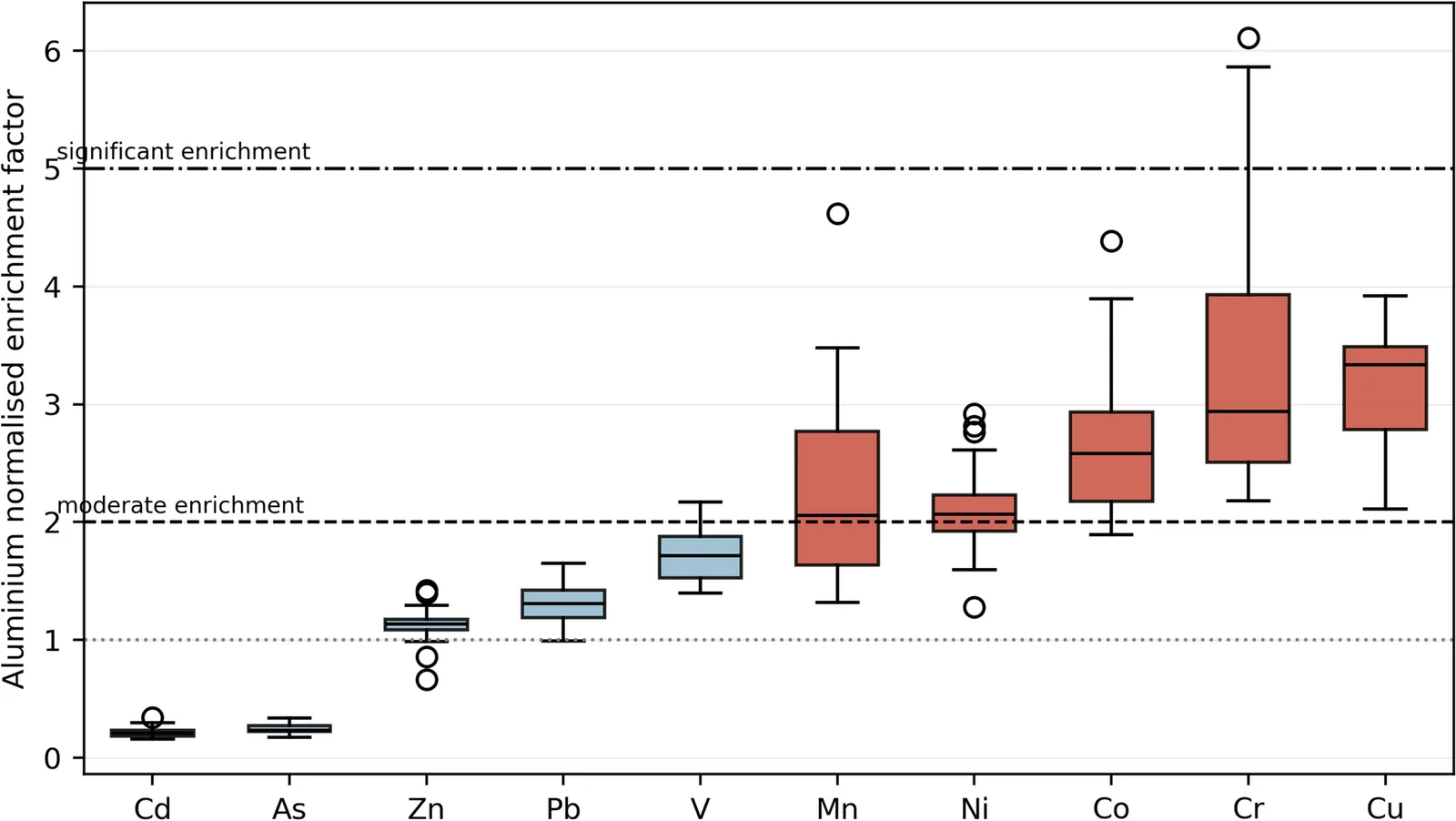
Aluminium normalised enrichment factors for ten elements. Dashed lines indicate the descriptive thresholds of 2 and 5. Values above 2 indicate a departure from the average shale element to Al ratio, not proof of anthropogenic contamination

Enrichment factors provided a similar but more qualified interpretation (Fig. 3). Chromium ranged from 2.2 to 6.1, Cu from 2.1 to 3.9, Co from 1.9 to 4.4, Mn from 1.3 to 4.6, Ni from 1.3 to 2.9 and V from 1.4 to 2.2 relative to average shale. Lead ranged from 0.99 to 1.65, Zn from 0.66 to 1.42, Cd from 0.16 to 0.34, As from 0.17 to 0.34 and Hg from 0.07 to 0.16. Geo-accumulation index values for Pb, Zn, As and Cd were below zero at every site. Combined, these results are consistent with predominantly lithogenic control and provide no clear evidence for strong enrichment of the canonical anthropogenic markers. They do not establish that all Cr, Cu, Ni or Co is natural, because both the reference composition and the aluminium normalisation assumption remain influential, and an enrichment factor between 2 and 5 is as compatible with a mafic parent material as with moderate anthropogenic addition.

### Depth associated depositional gradient

Water depth was positively associated with Al (ρ = 0.64, *p* = 0.003), Cr (ρ = 0.56, *p* = 0.012), Ni (ρ = 0.59, *p* = 0.007), Cu (ρ = 0.58, *p* = 0.009), Pb (ρ = 0.59, *p* = 0.008) and the mean probable effect concentration quotient (ρ = 0.61, *p* = 0.006), with *n* = 19 sites for which depth was recorded. These associations are moderate in strength, and their interpretation remains inferential rather than demonstrated. Grain size, total organic carbon, sedimentation rate and hydrodynamic conditions were not measured, so water depth cannot be shown to act as a direct control on element accumulation. Depth is more plausibly a proxy for depositional energy, with deeper sites occupying lower energy settings that accumulate a finer, matrix rich particle assemblage. The result is therefore described as a depth associated depositional gradient, and the underlying mechanism remains untested.

### Sediment quality guideline screening

**Fig. 4:**
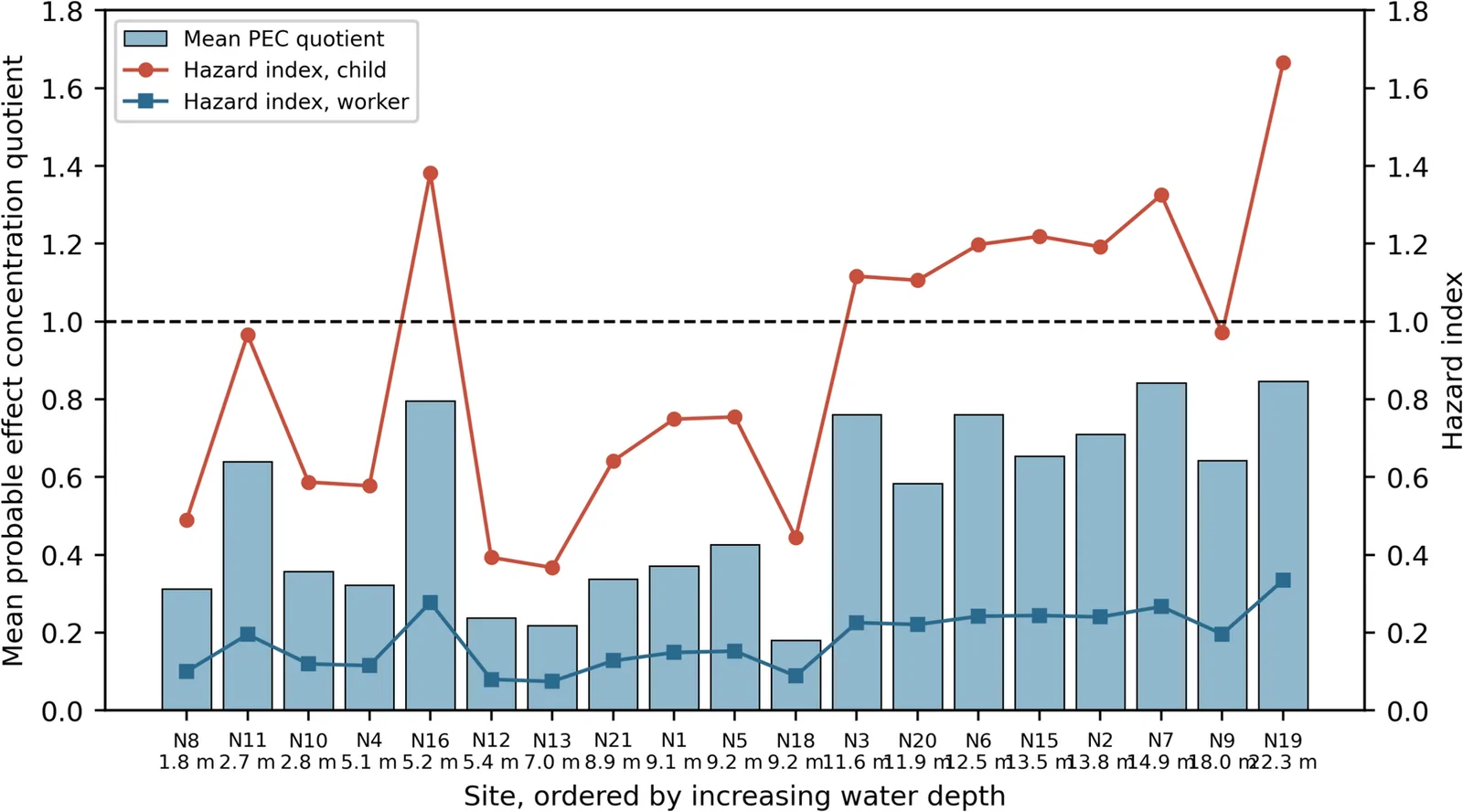
Mean probable effect concentration quotient by site, ordered by increasing water depth, with deterministic hazard indices for the adult maintenance worker and child incidental contact scenarios on the secondary axis. The dashed line marks a hazard index of one. The figure compares exposure scenarios and does not represent observed population risk

Guideline screening identified potential concerns for Cr and Ni despite limited evidence of enrichment in the canonical anthropogenic markers (Table 3). Chromium exceeded the TEC at all 21 sites and the PEC at 14 sites. Nickel exceeded the TEC at 20 sites and the PEC at 13. Copper exceeded the TEC at 15 sites but the PEC at only two. Arsenic, Cd, Pb, Zn and quantified Hg did not exceed their TEC at any site. The mean PEC quotient ranged from 0.18 at N18 to 0.85 at N19, with a mean of 0.52, and exceeded 0.5 at 11 of 21 sites (Fig. 4). The elements contributing most to this screening signal were Cr and Ni, which also showed the strongest matrix associated spatial patterns. This combination supports a separation between source oriented enrichment and potential ecological hazard (Long et al., 1995; MacDonald et al., 2000; Oze et al., 2004). It is important to state the limits of this result explicitly. Guideline exceedance is not evidence of contamination, because the consensus benchmarks are indifferent to element provenance and the source diagnostics reported above point to matrix control. Neither is it evidence of toxicity, because the quotient is a probability based screening metric derived from a broader effects database and does not account for local speciation, particle size, organic carbon or sulphide binding. What the result establishes is a screening–level trigger for site specific verification, not a demonstrated ecological effect at Nandoni.

**Table 3:** Sediment quality screening results and sensitivity of composite contamination indices to the selected external reference background. Both backgrounds were applied to the same six elements (Cd, Cu, Cr, Ni, Pb and Zn)

Metric	Value
Sites exceeding Cr threshold effect concentration	21 of 21
Sites exceeding Cr probable effect concentration	14 of 21
Sites exceeding Ni threshold effect concentration	20 of 21
Sites exceeding Ni probable effect concentration	13 of 21
Sites exceeding Cu threshold effect concentration	15 of 21
Sites exceeding Cu probable effect concentration	2 of 21
Sites exceeding As, Cd, Pb, Zn or Hg threshold effect concentration	0 of 21
Mean probable effect concentration quotient	0.18–0.85
Pollution load index, average shale background	0.20–1.06 (1 site above 1)
Pollution load index, pre–industrial background	1.06–5.44 (21 sites above 1)
Potential ecological risk index, average shale background	9.6–53.4 (all low risk)
Potential ecological risk index, pre–industrial background	76.7–430.3 (13 moderate, 8 high)

### Human health screening and receptor dependence

For the adult maintenance worker scenario, the hazard index ranged from 0.073 at N14 to 0.335 at N19 and remained below one at all sites (Table 4, Fig. 4). Ingestion accounted for 86.2% and dermal contact for 13.8% of the summed hazard index, the dermal share being highest for Cr, for which the low gastrointestinal absorption fraction raises the dermal hazard quotient relative to the oral one. Mean hazard quotients were greatest for Co (0.062), followed by Cr (0.054), Mn (0.036) and V (0.017). For the child incidental contact scenario, the hazard index ranged from 0.360 to 1.665 and exceeded one at nine of 21 sites, with ingestion accounting for 93.7% of the total. Cobalt was again the largest contributor (0.333), followed by Cr (0.233), Mn (0.180) and V (0.090). The contrast between receptors is driven by the ratio of ingestion rate to body mass and by the exposure duration assumptions, and is therefore a property of the exposure model rather than a measure of differences in sediment toxicity. The summed incremental lifetime cancer risk ranged from 1.67 × 10^−5^ to 8.87 × 10^−5^ for the worker and from 1.55 × 10^−5^ to 8.20 × 10^−5^ for the child, with Cr and Ni contributing approximately 95% of the total in both scenarios. These estimates fall within the commonly used 10^−6^ to 10^−4^ risk management range, although no site fell below 10^−5^. The Cr(III) sensitivity calculation quantifies how much of this depends on the oxidation state assumption. Treating all measured Cr as Cr(III) rather than Cr(VI) reduced the worker hazard index to 0.048 to 0.251 and the child hazard index to 0.253 to 1.301, which reduced the number of sites with a child hazard index above one from nine to three. The same substitution reduced incremental lifetime cancer risk to 6.90 × 10^−6^ to 4.69 × 10^−5^ for the worker and 6.38 × 10^−6^ to 4.34 × 10^−5^ for the child, a reduction of ~ 47%, and placed four sites below 10^−5^. Chromium speciation is therefore the single largest source of uncertainty in the health screening, and the two calculations should be read as bracketing the plausible range rather than as competing estimates. All values are modelled screening estimates under generic exposure parameters and are not measured occupational or community risk.

**Table 4:** Deterministic human health screening estimates for an adult maintenance worker scenario and a child incidental contact scenario across the 21 sites

Metric	Adult maintenance worker	Child, incidental contact
Hazard index, minimum	0.073	0.36
Hazard index, mean	0.182	0.906
Hazard index, maximum	0.335	1.665
Sites with hazard index above 1	0 of 21	9 of 21
Largest contributors	Co, then Cr, Mn and V	Co, then Cr, Mn and V
Incremental lifetime cancer risk, range	1.67 to 8.87 × 10^−5^	1.55–8.20 × 10^−5^
Dominant carcinogenic contributors	Cr and Ni (~ 95%)	Cr and Ni (~ 95%)

### Sensitivity to the selected background

**Fig. 5:**
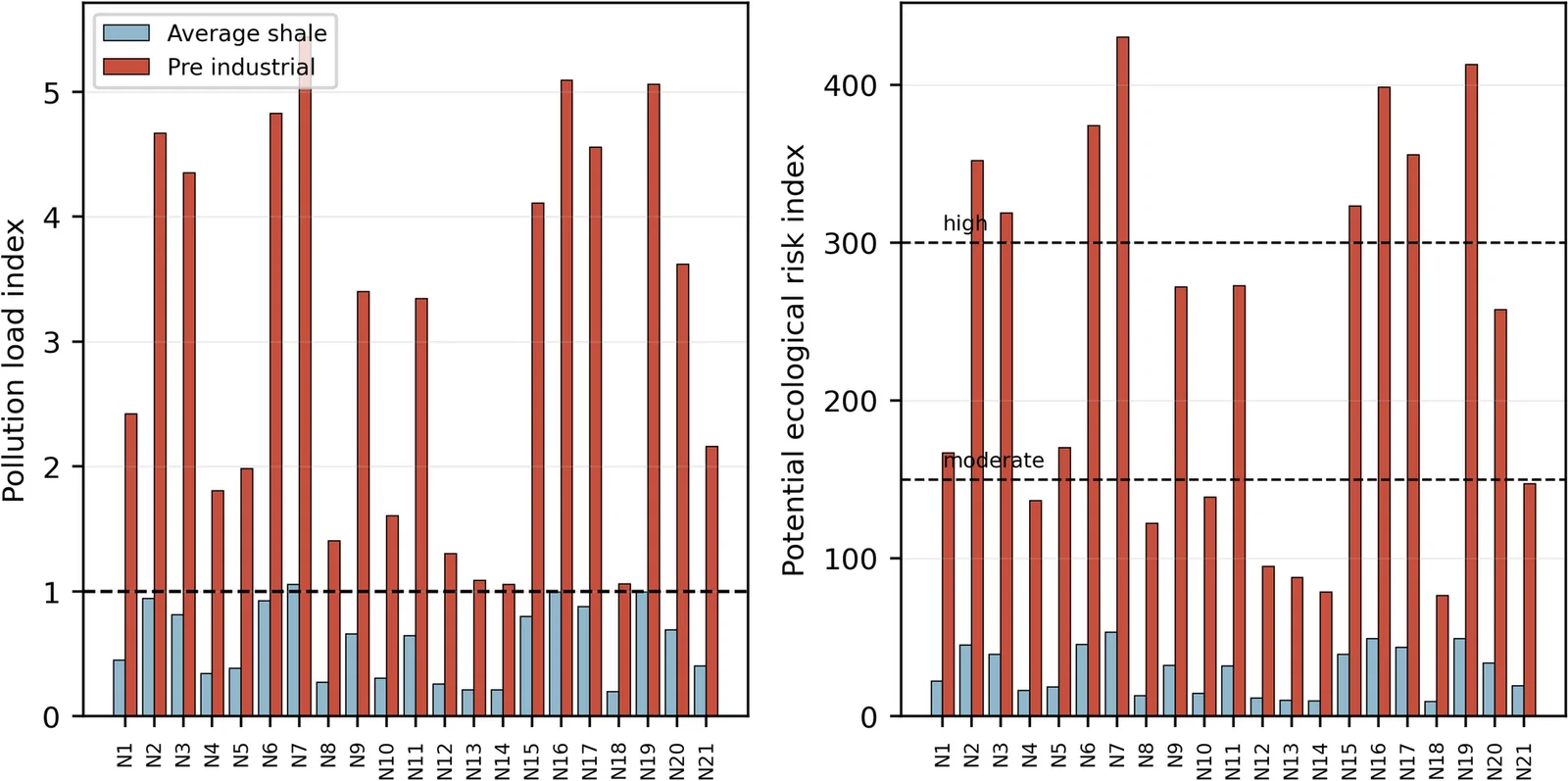
Sensitivity of the pollution load index and the potential ecological risk index to the selected external reference. Blue bars use average shale and red bars use the lower pre–industrial reference values reported by Sojka et al. (2022b). Dashed lines show conventional screening thresholds and should not be interpreted as site specific regulatory limits

Composite indices were highly sensitive to the selected external reference (Fig. 5). Both backgrounds were applied to the same 6 elements (Cd, Cu, Cr, Ni, Pb and Zn) to ensure that comparisons were made on a consistent basis. With average shale as the background, contamination factors exceeded one at 14 sites for Cr, 13 for Cu, 11 for Ni, five for Pb, two for Zn and none for Cd, and additionally at 15 sites for Co and site11 each for V and Mn. The pollution load index ranged from 0.26 to 1.35 (mean 0.76), exceeding one at 8 sites, and the potential ecological risk index ranged from 9.2 to 51.0 (mean 27.6), classifying all 21 sites as low risk. When the lower pre–industrial reference values reported by Sojka et al. (2022b) were applied to the same measured concentrations, the pollution load index ranged from 1.06 to 5.44 (mean 3.06) and exceeded one at all 21 sites, and the potential ecological risk index ranged from 76.7 to 430.3 (mean 237.5), classifying 8 sites as low, five as moderate and eight as high risk. The decomposition identifies the origin of this shift precisely (Table 5). For the pollution load index, the inflation factor is the geometric mean of the six element wise background ratios and equals 4.04 at every site without exception, because it does not depend on the measured concentrations at all. The apparent fourfold increase in pollution load index therefore carries no information about Nandoni sediment and is an arithmetic consequence of the background substitution alone. For the potential ecological risk index the mean increase of 209.9 units is attributable almost entirely to three elements: Ni contributes 42.4%, Cu 29.1% and Cr 27.2%, together 98.7% of the total change. These are the three elements for which the pre–industrial reference values (5, 6 and 5 mg kg^−1^, respectively) are most strongly discordant with the mafic and ultramafic influenced geology of the Soutpansberg catchment, where background Cr and Ni of several tens of mg kg^−1^ would be expected from the parent material alone. Cadmium moves in the opposite direction (– 0.7%) and Pb and Zn contribute 1.7% and 0.3%. The reclassification is therefore driven by the three elements whose assumed background is least defensible for this catchment, which is the clearest possible demonstration that the shift reflects methodological uncertainty in the reference composition and not a change in contamination or ecological risk. Neither external reference set is a substitute for a regional geochemical baseline derived from parent material, catchment soils, pre–impoundment sediment or screened reference sites.

**Table 5:** Decomposition of the change in composite indices on substituting the pre–industrial reference set for average shale. B1 is the average shale background and B2 the pre–industrial background (mg kg^−1^). The pollution load index inflation factor is the geometric mean of B1i/B2i across the six elements and is identical at every site. The potential ecological risk index contribution is Ti × Ci × (1/B2i – 1/B1i), averaged across sites and expressed as a percentage of the mean total change of 209.9 units

Element	B1 average shale	B2 pre–industrial	B1/B2	Mean ER, shale	Mean ER, pre–industrial	Mean change in ER	Share of total PERI change (%)
Cd	0.3	0.5	0.6	3.62	2.17	− 1.4	− 0.7
Cu	45	6	7.5	9.39	70.45	61.1	29.1
Cr	90	5	18.0	3.36	60.51	57.1	27.2
Ni	68	5	13.6	7.06	96.07	89.0	42.4
Pb	20	10	2.0	3.54	7.09	3.5	1.7
Zn	95	48	2.0	0.63	1.24	0.6	0.3
Total			4.04 (PLI inflation factor)	27.6	237.5	209.9	100.0

## Discussion

The primary finding is not whether Nandoni sediment is safe or contaminated, but that source attribution, ecological screening and exposure assessment address distinct questions and generate different spatial interpretations of the same data. The appropriate management response is therefore targeted verification of bioavailability and exposure rather than an automatic decision based on a single composite index. The source oriented evidence is internally coherent but must be stated as inference rather than demonstration. Strong associations with Al, relatively stable element to Al ratios, low Pb, Zn, Cd, As and Hg enrichment relative to average shale, and a shared spatial pattern are together consistent with predominantly lithogenic and depositional control. They do not demonstrate it. Grain size, mineralogy, total organic carbon, Fe and Mn oxide content, sediment provenance indicators and a local geochemical baseline were not measured, and without them lithogenic control remains plausible rather than established. Aluminium normalisation cannot separate natural fine particle transport from diffuse anthropogenic inputs that share the same transport pathway, and the low enrichment of Pb, Zn, Cd, As and Hg does not exclude diffuse anthropogenic inputs. What the data show is that such sources did not generate a large, independently varying signal in the sampled surface sediment. The contrast with other Limpopo systems is instructive and can be stated specifically. In the mining–influenced Olifants catchment, Dahms et al. (2017) reported trace element distributions that depart from detrital proportion, with enrichment concentrated at discrete points downstream of identifiable discharges rather than distributed along a depositional gradient. Along the urban–agricultural gradient described by Dalu et al. (2018), the anthropogenic markers Pb and Zn increased with urban land cover independently of the matrix elements, which is the decoupling signature absent at Nandoni. In the artisanal mining catchments reviewed by Duncan et al. (2018), Cd and Pb geo–accumulation values exceeded zero, whereas at Nandoni they are negative at every site. The Nandoni pattern, in which Cr, Ni and Cu rise and fall in fixed proportion to Al while Pb, Zn, Cd, As and Hg remain at or below crustal proportion, more closely resembles reports from mafic volcanic terrain such as the Cameroon Volcanic Line, where elevated Cr, Ni and Co in stream sediments track parent lithology rather than land use (Tiabou et al., 2025; Yiika et al., 2024). Ecological screening benchmarks, by contrast, evaluate biological risk independently of provenance and cannot by themselves indicate whether lithogenic weathering or external input produced the observed concentrations. Chromium exceeded the PEC at 14 sites and Ni at 13, and the mean PEC quotient exceeded 0.5 at 11 sites. These values indicate an elevated probability of effects within the benchmark derivation dataset. They do not demonstrate toxicity at Nandoni, because total concentrations cannot resolve oxidation state, mineral host, pH, redox conditions, organic carbon, sulphide binding or grain size, and because the environmental behaviour and toxicity of Cr depend strongly on whether it occurs as Cr(III) or Cr(VI) (Oze et al., 2004).

The receptor comparison exposes a limitation of reporting only an occupational scenario. The worker model produced hazard indices below one at every site, whereas the child incidental contact model exceeded one at nine sites. The contrast is a property of the exposure assumptions, particularly sediment ingestion relative to body mass, and not a measure of differences in sediment toxicity (Sojka et al., 2022b; US EPA, 2011, 2026; Varol, 2020). Cobalt, Cr, Mn and V contributed most to the child index. The child scenario is deliberately conservative and should be read as a sensitivity analysis for an accessible shoreline rather than as a characterisation of an observed population. Shoreline access, contact frequency and actual sediment ingestion at Nandoni reservoir have not been established, and the generic parameters used here may overstate exposure if marginal sediment is seldom exposed or seldom visited by children. A hazard index above one under these assumptions is a trigger for refinement of exposure and toxicity information, not a diagnosis of harm. The chromium oxidation state assumption is the largest single source of uncertainty in the health screening and should be treated as such rather than as a minor caveat. Assigning all total Cr to Cr(VI) maximises both the hazard index and the cancer risk, and the sensitivity calculation reported above shows the consequence directly: under a Cr(III) assumption the number of sites with a child hazard index above one falls from nine to three and cancer risk falls by approximately 47%. Reduced, organic rich profundal sediment derived from mafic parent material would be expected to favour Cr(III), so the true value probably lies nearer the lower bracket, but this cannot be asserted without speciation data. The use of generic oral toxicity values introduces further uncertainty, because chemical form and exposure route both affect them, and no validated dermal slope factors exist for the carcinogenic elements considered (Oze et al., 2004; US EPA, 2026). The estimated risks therefore justify speciation and bioavailability measurement before any site–specific health conclusion is drawn, and they do not support a claim that residents or workers carry an established excess risk of cancer.

Background choice had the widest effect on the management narrative, as the same measured concentrations were classified as low risk against average shale and as moderate to high risk against the lower pre–industrial reference set. This is a property of ratio based indices and not evidence that the reservoir changed between calculations, a point the decomposition in Table 5 establishes quantitatively: the pollution load index inflation factor of 4.04 is constant across all sites and independent of the measured data, and 98.7% of the change in the potential ecological risk index is produced by the 3 elements whose assumed pre–industrial background is least compatible with a mafic catchment. It follows that neither the pollution load index nor the potential ecological risk index can resolve contamination source or actual ecological risk in the absence of a defensible local background and of biological or bioavailability evidence. Reported in isolation, they express an assumption about geology as though it were a measurement of pollution. This is consistent with wider warnings that enrichment and pollution indices are distorted when generic backgrounds are transferred across geological regions (Birch, 2017; Burton, 2002; Coral et al., 2022; Reimann & de Caritat, 2005). A defensible monitoring programme should state the selected background, its geological basis and its uncertainty, and should pair composite indices with element to reference normalisation, spatial pattern, sediment characteristics and biological evidence.

The sampling was confined to a single occasion at the end of one dry season, with one composite sample per site, so neither seasonal variability nor within site heterogeneity is characterised, and very shallow or obstructed marginal areas are under represented. The supporting sediment properties that govern partitioning and bioavailability, namely grain size, total organic carbon, acid volatile sulphide, pore water chemistry, mineralogy and Fe and Mn oxide content, were not measured, which precludes multivariable modelling and restricts the guideline comparison to a screening role. The digestion recovers pseudo–total concentrations and the analysis does not resolve chemical speciation, so the chromium oxidation state, which the sensitivity analysis identifies as the dominant uncertainty in the health screening, remains unknown. Furthermore, no locally derived geochemical baseline exists for the Soutpansberg catchment, so both backgrounds used here are external comparators and the resulting contamination indices remain screening classifications rather than evidence of anthropogenic enrichment.

We therefore recommend four follow up measurements, were a local geochemical baseline, developed from catchment soils over the principal lithologies, from pre–impoundment or deep core sediment, and from screened reference sites, because without it every contamination index reported for this catchment, including those reported here, remains background limited rather than data limited. The chromium speciation together with operationally defined bioavailability, by sequential extraction and by Cr(VI) specific determination, since this single measurement would collapse the largest uncertainty in both the ecological and the health screening. We recommend a routine measurement of grain size and total organic carbon, with acid volatile sulphide and simultaneously extracted metals where feasible, which would allow the depth associated gradient to be tested mechanistically and would support normalisation of the guideline comparison. Lastly, biological validation through paired benthic macroinvertebrate sampling and whole sediment toxicity testing at sites spanning the observed PEC quotient range, which is the only line of evidence capable of converting a screening exceedance into a demonstrated effect. Verification of shoreline accessibility and of actual contact behaviour would, in parallel, allow the child exposure scenario to be replaced with locally parameterised estimates.

## Conclusions

Surface sediment across Nandoni Reservoir shows a spatially coherent, predominantly matrix associated elemental pattern. Chromium, Ni, Cu and Pb tracked Al closely, while Pb, Zn, Cd, As and Hg showed little evidence of enrichment relative to average shale. These results are consistent with predominantly lithogenic and depositional control. They do not constitute a demonstrated source attribution, which would require grain size, mineralogy, total organic carbon, provenance indicators and a local geochemical baseline, and the absence of a strong, independently varying signal from the canonical anthropogenic markers should not be read as the absence of anthropogenic influence. At the same time, Cr exceeded the consensus PEC at 14 of 21 sites and Ni at 13, and the mean PEC quotient exceeded 0.5 at 11 sites. Deterministic child incidental contact screening produced hazard indices above one at 9 sites, falling to three when Cr was treated as Cr(III), whereas the adult worker scenario remained below one throughout. Cancer risk estimates remained within the commonly used management range but exceeded 10^–5^ under the conservative Cr(VI) assumption. Each of these is a screening level result that depends on generic benchmarks and conservative exposure parameters, and none of them establishes confirmed ecological toxicity or a human health effect. What the study establishes is potential concern requiring verification. Composite indices moved from low to moderate or high classification when an alternative external background was substituted, a shift produced almost entirely by Ni, Cu and Cr and by the background values assumed for them rather than by the sediment. Nandoni reservoir therefore shows why provenance, ecological screening and exposure assessment must be kept analytically separate. Geogenic elevation does not render a potential hazard irrelevant, and generic background indices should not be used alone to trigger source–control decisions in geologically heterogeneous catchments, particularly where a locally derived baseline is unavailable.

## Supplementary Information

Below is the link to the electronic supplementary material.

Supplementary file1 (DOCX 19 KB)

## Data Availability

All data supporting the findings of this study are presented in the article.

## References

[CR1] Barker, O. B., Brandl, G., Callaghan, C. C., Eriksson, P. G., & van der Neut, M. (2006). The Soutpansberg and Waterberg groups and the Blouberg formation. In M. R. Johnson, C. R. Anhaeusser, & R. J. Thomas (Eds.), *The geology of South Africa* (pp. 301–318). Geological Society of South Africa and Council for Geoscience.

[CR2] Barnhoorn, I. E. J., Bornman, M. S., Jansen van Rensburg, C., & Bouwman, H. (2009). DDT residues in water, sediment, domestic and indigenous biota from a currently DDT–sprayed area. *Chemosphere,**77*(9), 1236–1241. 10.1016/j.chemosphere.2009.09.037 19782384

[CR3] Bing, H., Liu, Y., Huang, J., Tian, X., Zhu, H., & Wu, Y. (2022). Dam construction attenuates trace metal contamination in water through increased sedimentation in the Three Gorges Reservoir. *Water Research,**217*, 118419. 10.1016/j.watres.2022.118419 35413561

[CR4] Birch, G. F. (2017). Determination of sediment metal background concentrations and enrichment in marine environments: A critical review. *Science of the Total Environment,**580*, 813–831. 10.1016/j.scitotenv.2016.12.028 27986324

[CR5] Burton, G. A., Jr. (2002). Sediment quality criteria in use around the world. *Limnology,**3*(2), 65–75. 10.1007/s102010200006

[CR6] Canpolat, Ö., Varol, M., Okan, Ö. Ö., & Eriş, K. K. (2022). Sediment contamination by trace elements and the associated ecological and health risk assessment: A case study from a large reservoir, Turkey. *Environmental Research,**204*, 112145. 10.1016/j.envres.2021.112145 34597661

[CR7] Coral, K. C., Rodríguez-Romero, A., Tovar-Sánchez, A., Ruiz-Gutiérrez, G., & Fuente, J. R. V. (2022). Geochemical baseline establishment, contamination level and ecological risk assessment of metals and As in the Limoncocha lagoon sediments, Ecuadorian Amazon region. *Journal of Soils and Sediments,**22*(1), 293–315. 10.1007/s11368-021-03084-w

[CR8] Dahms, S., Baker, N. J., & Greenfield, R. (2017). Ecological risk assessment of trace elements in sediment: A case study from Limpopo, South Africa. *Ecotoxicology and Environmental Safety,**135*, 106–114. 10.1016/j.ecoenv.2016.09.036 27721124

[CR9] Dalu, T., Wasserman, R. J., Wu, Q., Froneman, W. P., & Weyl, O. L. F. (2018). River sediment metal and nutrient variations along an urban–agriculture gradient in an arid austral landscape: Implications for environmental health. *Environmental Science and Pollution Research,**25*(3), 2842–2852. 10.1007/s11356-017-0550-6 29143261

[CR10] Dong, J., Xia, X., Liu, Z., Zhang, X., & Chen, Q. (2019). Variations in concentrations and bioavailability of heavy metals in rivers during sediment suspension–deposition events induced by dams. *Journal of Soils and Sediments,**19*(1), 403–414. 10.1007/s11368-018-2071-2

[CR11] Duncan, A. E., de Vries, N., & Nyarko, K. B. (2018). Assessment of heavy metal pollution in the sediments of the River Pra and its tributaries. *Water, Air, and Soil Pollution,**229*(8), 272. 10.1007/s11270-018-3899-8 PMC6096550 30147192

[CR12] Edokpayi, J. N., Nkhumeleni, M., Enitan-Folami, A. M., & Olaniyi, F. C. (2022). Water quality assessment and potential ecological risk of trace metals in sediments of selected rivers in Vhembe District, South Africa. *Physics and Chemistry of the Earth, Parts a/b/c,**126*, 103111. 10.1016/j.pce.2022.103111

[CR13] Eggleton, J., & Thomas, K. V. (2004). A review of factors affecting the release and bioavailability of contaminants during sediment disturbance events. *Environment International,**30*(7), 973–980. 10.1016/j.envint.2004.03.001 15196845

[CR14] Frémion, F., Bordas, F., Mourier, B., Lenain, J. F., Kestens, T., & Courtin-Nomade, A. (2016). Influence of dams on sediment continuity: A study case of natural metallic contamination. *Science of the Total Environment,**547*, 282–294. 10.1016/j.scitotenv.2015.12.149 26789366

[CR15] Håkanson, L. (1980). An ecological risk index for aquatic pollution control: A sedimentological approach. *Water Research,**14*(8), 975–1001. 10.1016/0043-1354(80)90143-8

[CR16] Long, E. R., MacDonald, D. D., Smith, S. L., & Calder, F. D. (1995). Incidence of adverse biological effects within ranges of chemical concentrations in marine and estuarine sediments. *Environmental Management,**19*(1), 81–97. 10.1007/BF02472006

[CR17] MacDonald, D. D., Ingersoll, C. G., & Berger, T. A. (2000). Development and evaluation of consensus–based sediment quality guidelines for freshwater ecosystems. *Archives of Environmental Contamination and Toxicology,**39*(1), 20–31. 10.1007/s002440010075 10790498

[CR18] Madilonga, R. T., Edokpayi, J. N., Volenzo, E. T., Durowoju, O. S., & Odiyo, J. O. (2021). Water quality assessment and evaluation of human health risk in Mutangwi River, Limpopo Province, South Africa. *International Journal of Environmental Research and Public Health,**18*(13), 6765. 10.3390/ijerph18126675 PMC8296923 34202418

[CR19] Müller, G. (1969). Index of geoaccumulation in sediments of the Rhine River. *GeoJournal,**2*, 108–118.

[CR20] Nawrot, N., Wojciechowska, E., Matej-Łukowicz, K., Walkusz-Miotk, J., & Pazdro, K. (2021). Trace metal contamination of bottom sediments: A review of analytical methods, pollution sources and environmental risk assessment. *Minerals,**11*(8), 872. 10.3390/min11080872

[CR21] Oze, C., Fendorf, S., Bird, D. K., & Coleman, R. G. (2004). Chromium geochemistry in serpentinized ultramafic rocks and serpentine soils from the Franciscan Complex of California. *American Journal of Science,**304*(1), 67–101. 10.2475/ajs.304.2.67

[CR22] Reimann, C., & de Caritat, P. (2005). Distinguishing between natural and anthropogenic sources for elements in the environment: Regional geochemical surveys versus enrichment factors. *Science of the Total Environment,**337*(1–3), 91–107. 10.1016/j.scitotenv.2004.06.011 15626382

[CR23] Rudnick, R. L., & Gao, S. (2003). Composition of the continental crust. In H. D. Holland & K. K. Turekian (Eds.), *Treatise on geochemistry* (Vol. Vol. 3, pp. 1–64). Elsevier.

[CR24] Schropp, S. J., Lewis, F. G., Windom, H. L., Ryan, J. D., Calder, F. D., & Burney, L. C. (1990). Interpretation of metal concentrations in estuarine sediments of Florida using aluminium as a reference element. *Estuaries,**13*(3), 227–235. 10.2307/1351913

[CR25] Sigue, C., Yewong, N. E., Yiika, L. P., Tiabou, A. F., Simou, A., Liyouck, P. R., Ndema, M. J. L., & Yen, N. V. (2025). Assessment of contamination, sources and health risks of potentially hazardous elements in surface sediment of Dibang Cameroon. *Discover Environment,**3*, 165. 10.1007/s44274-025-00363-y

[CR26] Shibambu, T. H., Kahler, D. M., Rose, K. C., Riddell, E., Oyebanjo, O. M., & Edokpayi, J. N. (2025). Comprehensive monitoring of the spatiotemporal variation of water quality and its associated human health risks in Luvuvhu River Catchment, Vhembe Biosphere Reserve South Africa. *Scientific Reports,**15*, 36319. 10.1038/s41598-025-20228-x PMC12534464 41107308

[CR27] Sojka, M., Jaskuła, J., Barabach, J., Ptak, M., & Zhu, S. (2022a). Heavy metals in lake surface sediments in protected areas in Poland: Concentration, pollution, ecological risk, sources and spatial distribution. *Scientific Reports,**12*, 15006. 10.1038/s41598-022-19298-y PMC9440085 36056130

[CR28] Sojka, M., Ptak, M., Jaskuła, J., & Krasniqi, V. (2022b). Ecological and health risk assessments of heavy metals contained in sediments of Polish dam reservoirs. *International Journal of Environmental Research and Public Health,**20*(1), 324. 10.3390/ijerph20010324 PMC9819632 36612645

[CR29] Sojka, M., Siepak, M., Jaskuła, J., & Wicher-Dysarz, J. (2018). Heavy metal transport in a river–reservoir system: A case study from Central Poland. *Polish Journal of Environmental Studies,**27*(4), 1725–1734.

[CR30] Sutherland, R. A. (2000). Bed sediment–associated trace elements in an urban stream, Oahu Hawaii. *Environmental Geology,**39*(6), 611–627. 10.1007/s002540050473

[CR31] Tiabou, A. F., Esukac, C. N., Mbowou Ngantche, I. F., Yiika, L. P., Wadgua, B. P., & Agyingi, C. M. (2025). Spatial distribution, contamination characteristics and eco–risk assessment of toxic metals in stream sediments of Njombe-Penja Banana plain, Cameroon Volcanic Line. *Chemistry Africa,**8*, 619–639. 10.1007/s42250-024-01159-y

[CR32] Tomlinson, D. L., Wilson, J. G., Harris, C. R., & Jeffrey, D. W. (1980). Problems in the assessment of heavy–metal levels in estuaries and the formation of a pollution index. *Helgoländer Meeresuntersuchungen,**33*, 566–575. 10.1007/BF02414780

[CR33] Turekian, K. K., & Wedepohl, K. H. (1961). Distribution of the elements in some major units of the Earth’s crust. *Geological Society of America Bulletin,**72*(2), 175–192. 10.1130/0016-7606(1961)72[175:DOTEIS]2.0.CO;2

[CR34] US Environmental Protection Agency. (1989). *Risk assessment guidance for Superfund. Volume I: Human health evaluation manual, Part A* (EPA/540/1–89/002). Office of Emergency and Remedial Response.

[CR35] US Environmental Protection Agency. (2004). *Risk assessment guidance for Superfund. Volume I: Human health evaluation manual, Part E: Supplemental guidance for dermal risk assessment* (EPA/540/R/99/005).

[CR36] US Environmental Protection Agency. (2011). *Exposure factors handbook: 2011 edition* (EPA/600/R–09/052F). National Center for Environmental Assessment.

[CR37] US Environmental Protection Agency. (2026). *Regional screening levels for chemical contaminants at Superfund sites: Generic tables and user’s guide*. Retrieved September 8, 2026, from https://www.epa.gov/risk/regional-screening-levels-sls-generic-tables

[CR38] Varol, M. (2020). Environmental, ecological and health risks of trace metals in sediments of a large reservoir on the Euphrates River, Turkey. *Environmental Research,**187*, 109664. 10.1016/j.envres.2020.109664 32454309

[CR39] Wojciechowska, E., Nawrot, N., Walkusz-Miotk, J., Matej-Łukowicz, K., & Pazdro, K. (2019). Heavy metals in sediments of urban streams: Contamination and health risk assessment of influencing factors. *Sustainability,**11*(3), 563. 10.3390/su11030563

[CR40] Yiika, L. P., Ndema Mbongue, J. L., Suh, G. C., Nkemasong, J. A., Eseya Mengu, E. J., Fomekong, B. K., Sekem, S. G., & Kouankap, G. D. (2024). Distribution, sources and ecotoxicological assessment of potentially toxic metals in river sediments of Nkwen area, Cameroon Volcanic Line. *Water, Air, & Soil Pollution,**235*, 16. 10.1007/s11270-023-06830-7

[CR41] Zhu, H., Bing, H., Wu, Y., Zhou, J., Sun, H., Wang, J., & Wang, X. (2019). The spatial and vertical distribution of heavy metal contamination in sediments of the Three Gorges Reservoir determined by anti–seasonal flow regulation. *Science of the Total Environment,**664*, 79–88. 10.1016/j.scitotenv.2019.01.396 30743133

