# Supplementary material for: Predominantly lithogenic chromium and nickel elevate sediment guideline quotients and exposure screening in a subtropical African water supply reservoir
Source: Environ Geochem Health. 2026 Oct 26;48(16):665. doi: 10.1007/s10653-026-03558-1 (PMC13710582; doi:10.1007/s10653-026-03558-1)
Supplement: Supplementary file 1 [file 10653_2026_3558_MOESM1_ESM.docx]

# **Supplementary files**

**Table S1** Analytical quality control for Nandoni Reservoir surface sediment. Recovery and precision were assessed against certified reference material WQB-1 analysed in five replicates. Censoring is expressed as the percentage of the 21 stations at which the element fell below the limit of quantification

| **Element** | **Certified WQB-1 (mg kg^-1^)** | **Measured mean (mg kg^-1^)** | **SD** | **RSD (%)** | **Recovery (%)** | ***n* replicates** | **Stations quantified** | **Censored (%)** |
| --- | --- | --- | --- | --- | --- | --- | --- | --- |
| Al | 58 414 | 57 492.5 | 1 577.9 | 2.7 | 98.4 | 5 | 21 | 0 |
| Fe | 47 856 | 48 098.6 | 1 303.6 | 2.7 | 100.5 | 5 | 21 | 0 |
| Mn | 2 289 | 2 233.6 | 62.9 | 2.8 | 97.6 | 5 | 21 | 0 |
| Ba | 413.0 | 435.9 | 15.3 | 3.5 | 105.5 | 5 | 21 | 0 |
| Cr | 77.2 | 83.9 | 2.4 | 2.9 | 108.7 | 5 | 21 | 0 |
| V | 107.0 | 105.8 | 3.7 | 3.5 | 98.9 | 5 | 21 | 0 |
| Cu | 78.4 | 81.3 | 3.2 | 3.9 | 103.7 | 5 | 21 | 0 |
| Ni | 63.1 | 51.3 | 1.7 | 3.3 | 81.3 | 5 | 21 | 0 |
| Zn | 279.0 | 286.0 | 7.2 | 2.5 | 102.5 | 5 | 21 | 0 |
| Sr | 67.0 | 70.8 | 2.2 | 3.2 | 105.7 | 5 | 21 | 0 |
| Co | 18.1 | 15.2 | 0.5 | 3.4 | 83.7 | 5 | 21 | 0 |
| Pb | 85.0 | 85.6 | 3.0 | 3.5 | 100.8 | 5 | 21 | 0 |
| As | 23.0 | 25.2 | 0.6 | 2.4 | 109.5 | 5 | 21 | 0 |
| Sn | 5.9 | 6.4 | 0.2 | 3.3 | 107.8 | 5 | 21 | 0 |
| Se | 1.5 | 1.6 | 0.13 | 8.0 | 105.2 | 5 | 17 | 19 |
| Mo | 1.2 | 1.1 | 0.05 | 4.5 | 89.8 | 5 | 21 | 0 |
| Sb | 0.7 | 0.8 | 0.17 | 22.2 | 106.3 | 5 | 21 | 0 |
| Hg | 1.1 | 1.2 | 0.06 | 4.8 | 111.4 | 5 | 7 | 67 |
| Cd | 1.8 | 1.7 | 0.07 | 3.9 | 96.2 | 5 | 21 | 0 |
